# Adolescent dysmenorrhea revealing Herlyn-Werner-Wunderlich syndrome with endometriosis: A radiologic case report

**DOI:** 10.1016/j.radcr.2026.07.073

**Published:** 2026-08-14

**Authors:** Heru Haerudin, Harry Galuh Nugraha

**Affiliations:** Department of Radiology, Faculty of Medicine, Padjadjaran University, Dr. Hasan Sadikin Hospital, Bandung, Indonesia

**Keywords:** Herlyn-Werner-Wunderlich syndrome, Endometrioma, Dysmenorrhea, Magnetic resonance imaging, Müllerian duct anomaly, Obstructed hemivagina, Uterus didelphys

## Abstract

Herlyn-Werner-Wunderlich syndrome, also referred to as obstructed hemivagina with ipsilateral renal agenesis (OHVIRA) is a rare congenital urogenital anomaly resulting from abnormal development of the Müllerian and Wolffian ducts, classically characterized by uterus didelphys, obstructed hemivagina, and ipsilateral renal agenesis. It typically presents after menarche with nonspecific symptoms, often leading to delayed diagnosis and increased risk of complications such as hematometra and pelvic endometriosis. Radiologic imaging plays a key role in early detection and prevention of disease progression. A 16-year-old female presented with progressive dysmenorrhea for 1.5 years and irregular menstruation. Ultrasonography demonstrated uterus didelphys, a left adnexal cyst suggestive of endometrioma, and left renal agenesis. Magnetic resonance imaging (MRI) further delineated a noncommunicating left uterine unit with cervicovaginal atresia and hematometra, consistent with type 1.2 HWWS. MRI also confirmed a left ovarian endometrioma, showing T1 hyperintensity and T2 shading. CT urography further confirmed complete left renal and ureteral agenesis with preserved contralateral renal excretory function, completing the radiologic evaluation of the associated urinary tract anomaly. These multimodality imaging findings established the diagnosis and accurately defined the extent of the congenital anomaly and its associated complications. This case emphasizes the importance of early recognition of Herlyn-Werner-Wunderlich syndrome in adolescents with persistent dysmenorrhea and underscores the pivotal role of imaging. While ultrasonography is useful for initial assessment, MRI remains the gold standard for comprehensive evaluation. Early and accurate diagnosis is essential to guide management and prevent long-term complications, including endometriosis and infertility.

## Introduction

Herlyn-Werner-Wunderlich syndrome (HWWS), also known as obstructed hemivagina with ipsilateral renal agenesis (OHVIRA), is a rare congenital anomaly involving both Müllerian and Wolffian duct development. It is classically characterized by the triad of uterus didelphys, obstructed hemivagina, and ipsilateral renal agenesis [1,2]. Müllerian duct anomalies are relatively uncommon, with a reported prevalence of 0.8%-4% in the general population, while OHVIRA syndrome represents a particularly rare subset, with an estimated incidence of approximately 1 in 20,000 [3].

Clinically, the syndrome typically manifests after menarche, most commonly as progressive dysmenorrhea. However, the diagnosis is frequently delayed due to its nonspecific presentation and the presence of a patent contralateral hemivagina, which may give the impression of normal menstruation. Such delays can result in complications related to chronic outflow obstruction, including hematometra, pelvic infection, and long-term sequelae such as endometriosis, pelvic adhesions, and infertility [4,5].

In this report, we describe a 16-year-old adolescent presenting with progressive dysmenorrhea in whom delayed diagnosis led to the development of pelvic endometriosis. Initial evaluation with ultrasonography suggested a Müllerian duct anomaly and adnexal pathology, while magnetic resonance imaging (MRI) provided definitive characterization, confirming HWWS with associated hematometra and ovarian endometrioma. This case highlights the pivotal role of radiologic imaging, particularly MRI for accurate diagnosis and evaluation of associated complications in HWWS.

## Case presentation

A 16-year-old female presented with a 1.5-year history of progressively worsening dysmenorrhea. Initially, her menstrual cycles were regular; however, they gradually became irregular, occurring approximately every 3 months.

The patient reported intermittent left lower abdominal pain, particularly during menstruation. There were no associated symptoms such as nausea, vomiting, dysuria, abnormal vaginal discharge, or palpable abdominal mass. She attained menarche at the age of 14 years.

**Fig. 1:**
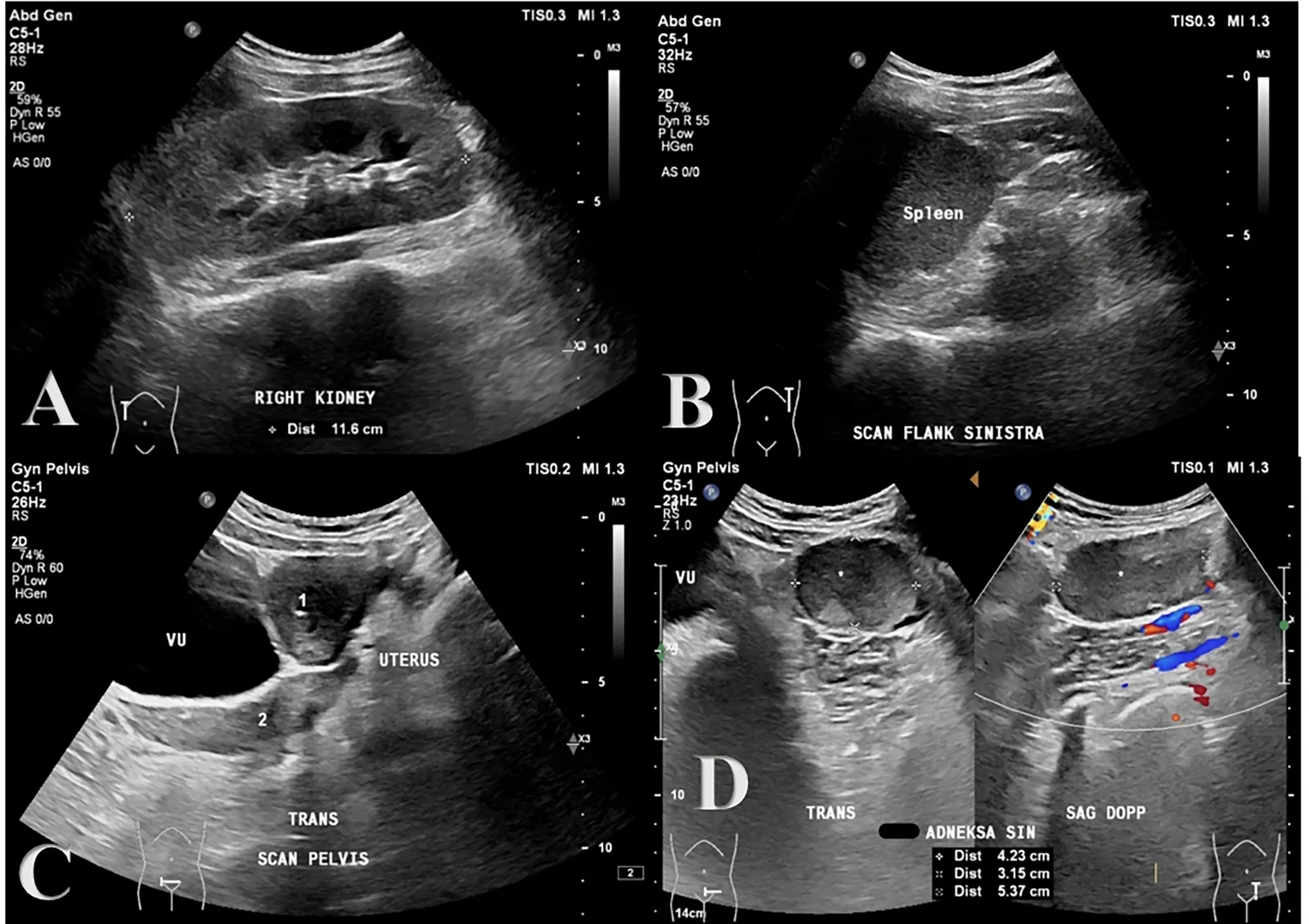
Transabdominal ultrasonography findings. (A) Normal right kidney. (B) Absence of the left kidney, consistent with renal agenesis. (C) Two separate uterine structures, suggestive of uterus didelphys. (D) Left adnexal cystic lesion demonstrating homogeneous low-level internal echoes with a ground glass appearance and peripheral echogenic foci, consistent with an endometrioma.

The patient underwent transabdominal ultrasonography as the initial imaging modality. Abdominal ultrasound demonstrated a normal right kidney, while no normal renal structure was identified in the left renal fossa, consistent with left renal agenesis. Pelvic ultrasound revealed the presence of 2 separate uterine structures, suggestive of uterus didelphys. In addition, a cystic lesion was identified in the left adnexal region, characterized by homogeneous low-level internal echoes producing a “ground-glass” appearance, with associated peripheral punctate echogenic foci, consistent with an endometriotic cyst (endometrioma). The lesion showed no significant internal vascularity on Doppler evaluation. These sonographic findings raised suspicion for a complex Müllerian duct anomaly with associated adnexal pathology (Fig. 1).

**Fig. 2:**
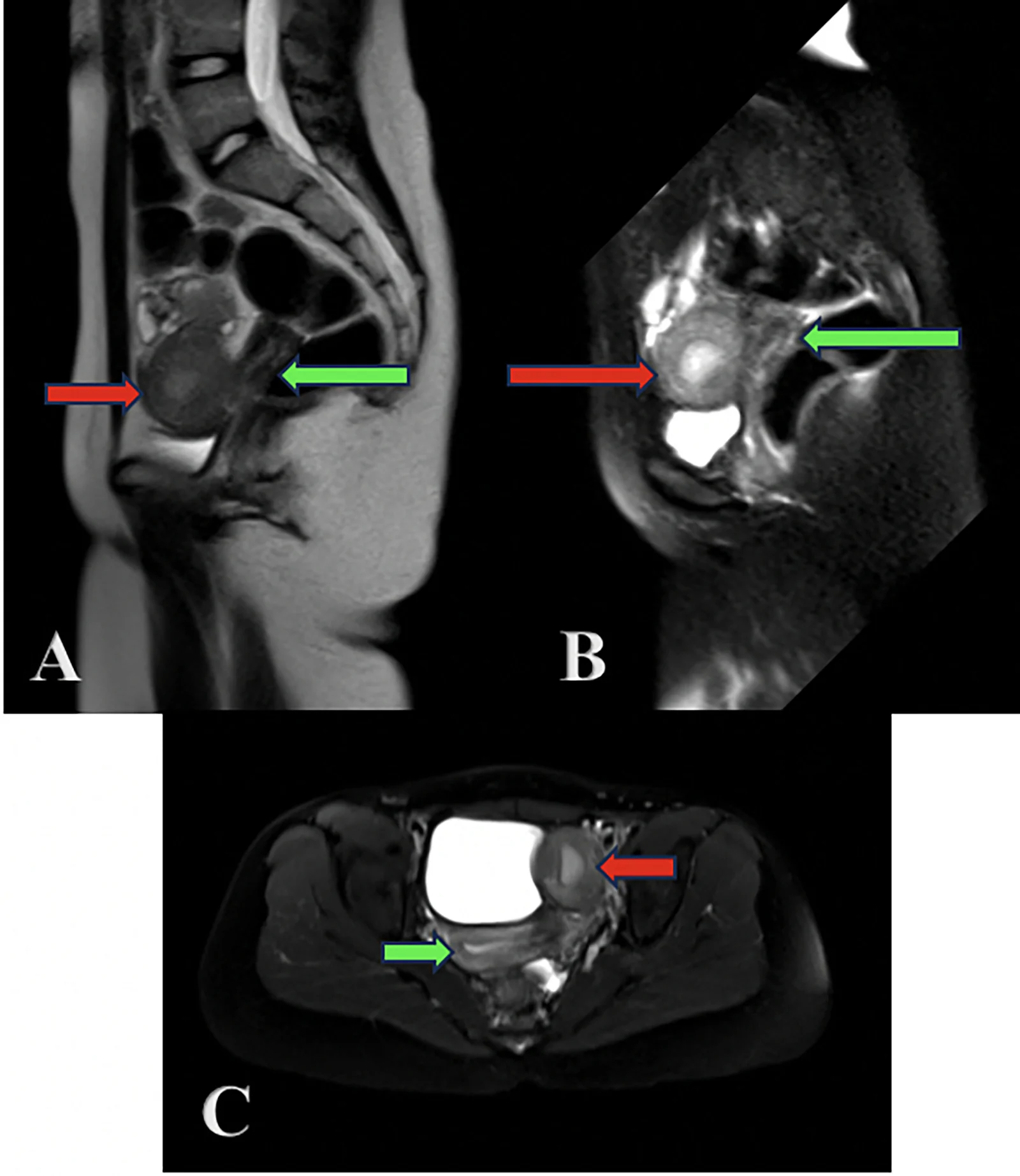
Pelvic MRI demonstrating features of Herlyn-Werner-Wunderlich syndrome with associated complications. (A) Sagittal T2-weighted image, (B) Sagittal T2-weighted fat-suppressed image, (C) Axial T2-weighted fat-suppressed image. All images demonstrate 2 separate uterine bodies consistent with uterus didelphys. The posterior structure represents the right uterus with a normal cervix and patent hemivagina (marked with a green arrow), without evidence of obstruction. The anterior enlarged structure represents the left uterus (marked with a red arrow), demonstrating absence of a visible cervix (cervical atresia), accompanied by marked distention of the endometrial cavity and no communication with the vaginal canal, consistent with obstructed hemivagina.

Pelvic magnetic resonance imaging (MRI) was subsequently performed to further characterize the Müllerian anomaly and to confirm the presence of associated complications. Sagital T2-weighted images, sagittal and axial T2-weighted fat-suppressed image demonstrated 2 completely separate uterine bodies, consistent with uterus didelphys. The right-sided uterus and cervix appeared normally formed and were connected to a patent hemivagina without evidence of obstruction. In contrast, the left uterine structure showed absence of a visible cervix, consistent with cervical atresia, accompanied by marked distention of the endometrial cavity without communication to the vagina, indicating complete obstruction of the left hemivagina. These findings are in keeping with obstructed hemivagina with ipsilateral renal anomaly, consistent with Herlyn-Werner-Wunderlich syndrome, specifically the cervicovaginal atresia subtype (Fig. 2).

**Fig. 3:**
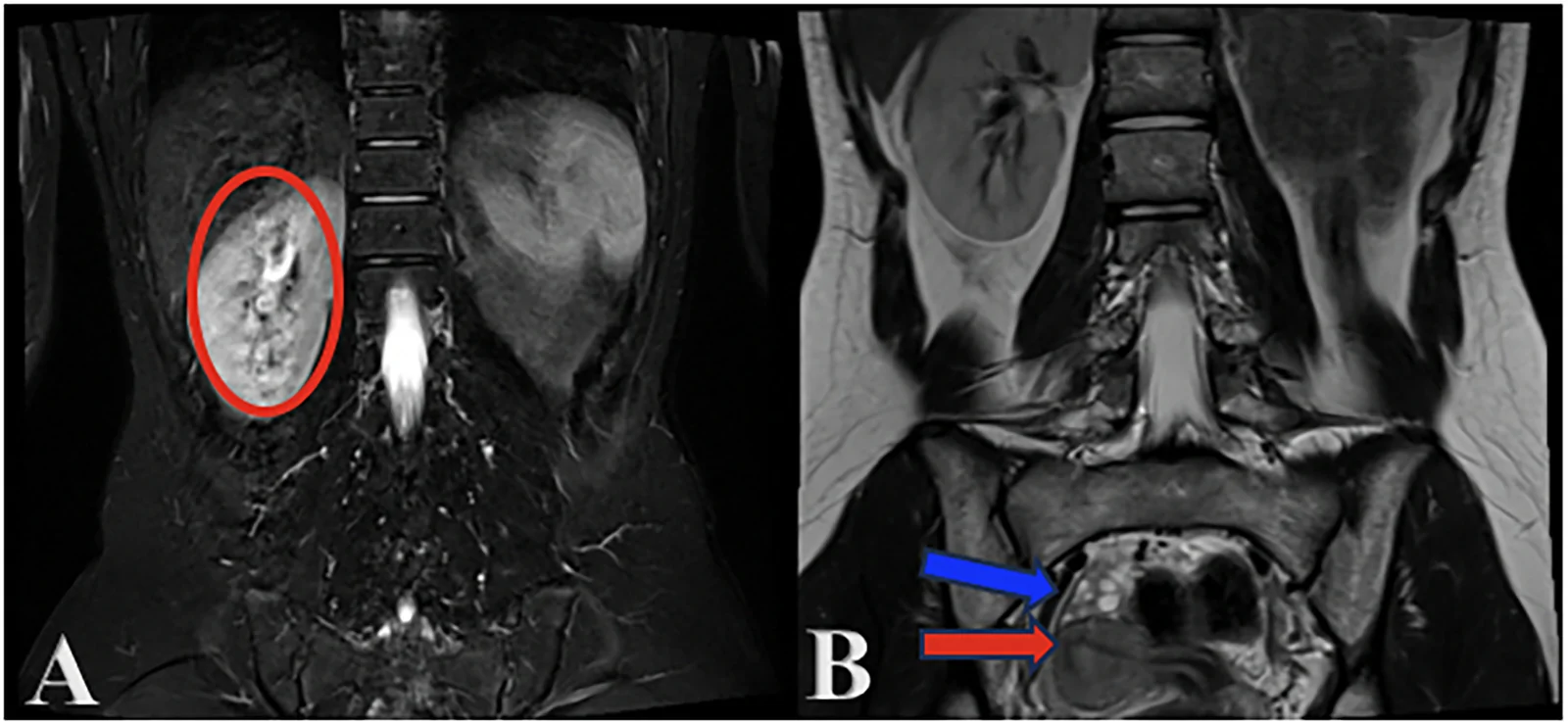
Abdominal MRI demonstrating renal anomaly associated with OHVIRA syndrome. (A) Coronal T2-weighted STIR image demonstrating a normal right kidney (marked with a red circle), with absence of the left kidney, consistent with left renal agenesis. (B) Coronal T2-weighted image showing the normal right kidney, absence of the left kidney, and visualization of the normal right uterus (marked with a red arrow) and ovary (marked with a blue arrow).

Coronal abdominal T2-weighted images demonstrated a normal right kidney, while the left kidney was not visualized, confirming left renal agenesis. The right adnexa appeared normal, with preservation of the right ovary (Fig. 3).

**Fig. 4:**
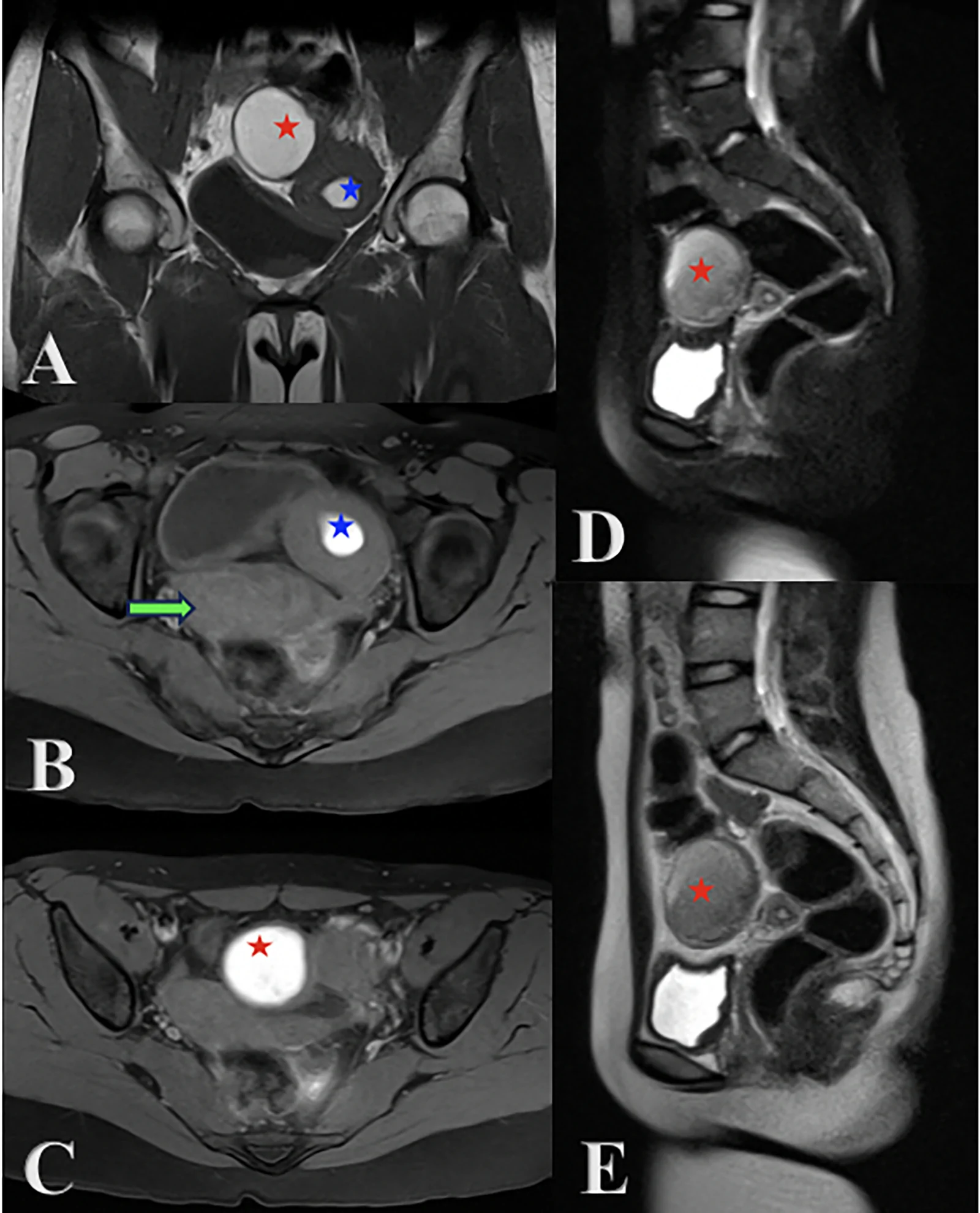
MRI features of hematometra and left ovarian endometrioma. (A) Coronal T1-weighted image, (B–C) Axial T1-weighted fat-suppressed images, and (D–E) Sagittal T2-weighted fat-suppressed and T2-weighted images. The left distended endometrial cavity (marked with a blue star) demonstrates high signal intensity on T1-weighted and T1 fat-suppressed images, consistent with hematometra. A cystic lesion in the left adnexal region (marked with a red star) demonstrates high signal intensity on T1-weighted images with corresponding low signal intensity on T2-weighted sequences, representing the characteristic shading sign, which reflects chronic blood products containing deoxyhemoglobin and methemoglobin, a hallmark feature of an ovarian endometrioma. The right uterine unit (marked with a green arrow) appears normal without evidence of obstruction.

Further evaluation with T1-weighted and T2-weighted pelvic sequences demonstrated marked distention of the left endometrial cavity with high signal intensity on T1-weighted and T1 fat-suppressed images, consistent with hematometra. Additionally, a cystic lesion was identified in the left adnexal region involving the left ovary, demonstrating high signal intensity on T1-weighted images with corresponding hypointensity on T2-weighted images, representing the shading sign characteristic of an ovarian endometrioma (Fig. 4).

**Fig. 5:**
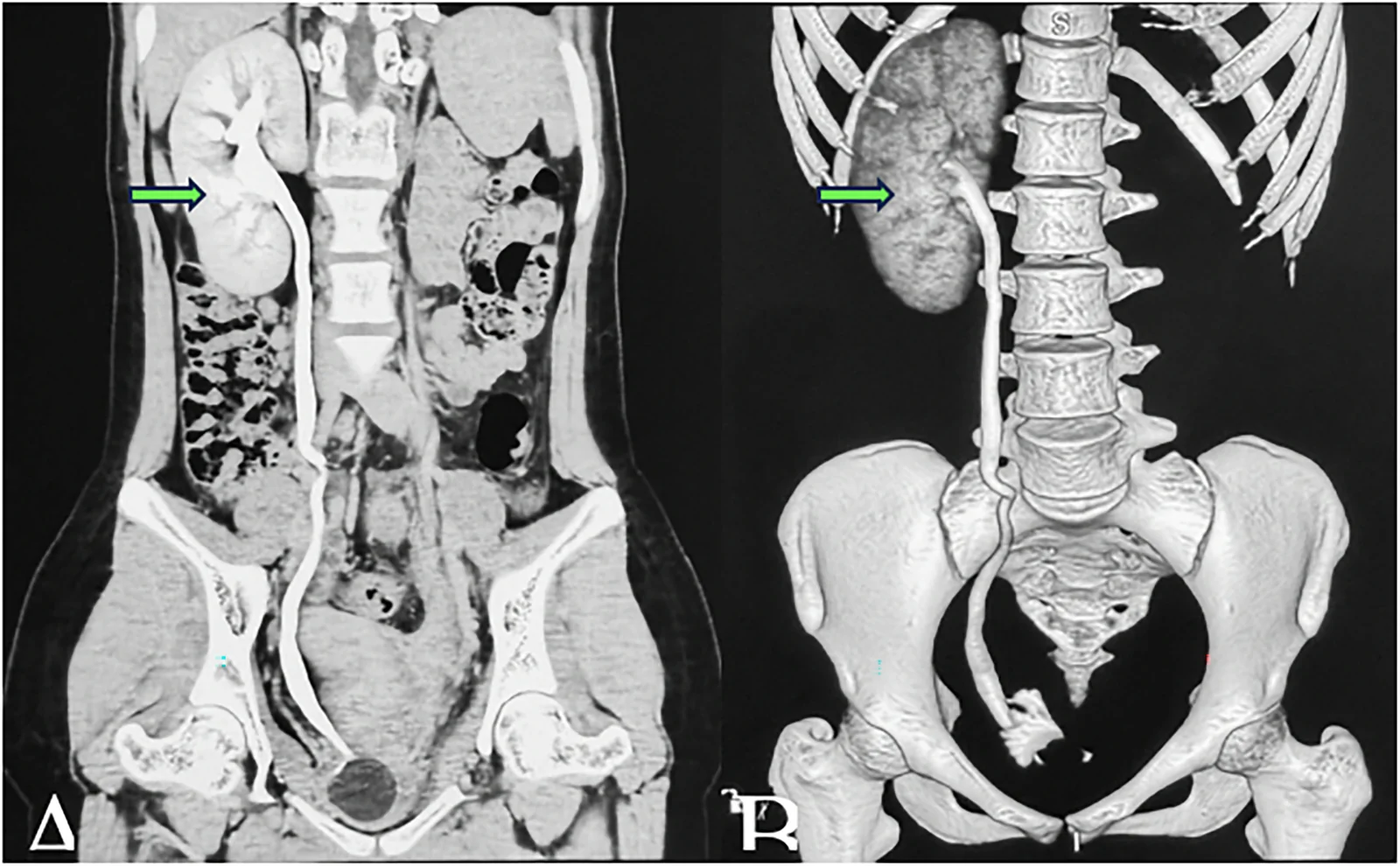
CT urography demonstrating the associated urinary tract anomaly. (A) Coronal contrast-enhanced CT urography (excretory phase) demonstrates complete absence of the left kidney and left ureter, consistent with left renal agenesis. The right kidney (marked with a green arrow) is normal in size and morphology, with homogeneous contrast enhancement and preserved excretory function, evidenced by opacification of the right renal collecting system and ureter. (B) Three-dimensional volume-rendered CT urography reconstruction confirms complete absence of the left kidney and ureter. The right kidney (marked with a green arrow) and ureter are normally developed, with preserved continuity of the right urinary tract extending to the urinary bladder.

To further evaluate the associated urinary tract anomaly, computed tomography (CT) urography in the excretory phase was performed. The examination demonstrated complete absence of the left kidney and left ureter, confirming left renal agenesis. The right kidney appeared normal in size, morphology, and contrast enhancement, with preserved excretory function and a normally opacified right ureter. These findings corroborated the MRI findings and further supported the diagnosis of Herlyn-Werner-Wunderlich syndrome by confirming the associated ipsilateral renal anomaly (Fig. 5).

**Fig. 6:**
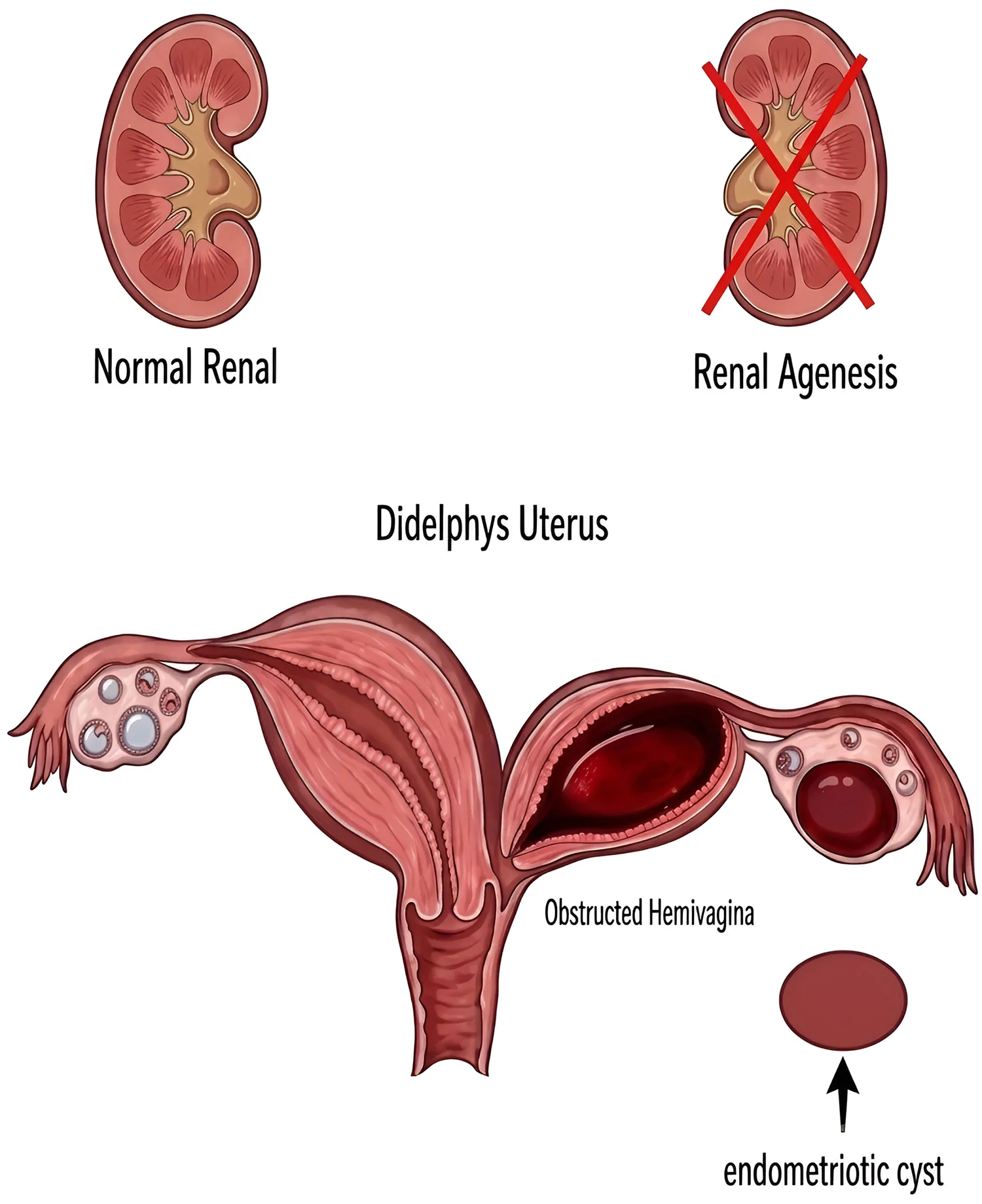
Schematic illustration of Herlyn-Werner-Wunderlich syndrome (classification 1.2) in the present case. Schematic illustration of Herlyn-Werner-Wunderlich syndrome demonstrating the classification 1.2 variant (cervicovaginal atresia without communicating uteri), corresponding to the present case. The diagram shows uterus didelphys with 2 completely separated uterine horns. The right uterine unit (the normal side) is connected to a patent cervix and vagina, allowing for normal menstrual outflow. In contrast, the left uterine unit demonstrates cervicovaginal atresia, resulting in complete outflow obstruction and hematometra. Ipsilateral (left-sided) renal agenesis is also depicted. Additionally, the left ovary demonstrates an endometrioma, likely secondary to chronic retrograde menstruation caused by longstanding obstruction. This schematic highlights the anatomical basis of the patient’s symptoms and the development of associated complications.

Based on the examination, her final diagnosis is a rare case of Herlyn-Werner-Wunderlich syndrome classification 1.2, also referred as cervicovaginal atresia without communicating uteri, characterized by characterized by uterus didelphys, obstructed hemivagina, ipsilateral renal agenesis, and associated endometrioma. The doctors had recommended surgery but she chose conservative treatment over surgery. Therefore, dienogest was administered for sympomatic treatment, particularly pain. Her symptoms improve after having dienogest. The summary findings in the present case was summarized in Figure 6.

## Discussion

Uterus didelphys has an estimated incidence ranging from 1 in 2000 to 1 in 28,000 women, and is associated with renal agenesis in up to 43% of cases. In contrast, unilateral renal agenesis itself may be accompanied by genital tract anomalies in 25%-50% of affected individuals [6,7].

Herlyn-Werner-Wunderlich syndrome typically presents after menarche with cyclic pelvic pain due to hematocolpos from an obstructed hemivagina; however, diagnosis is often delayed because normal menstruation from the contralateral patent hemivagina can obscure the condition. Such delay increases the risk of complications, particularly endometriosis secondary to chronic retrograde menstruation, as seen in this case [8].

Tong et al. reported that the incidence of pelvic endometriosis in patients with HWWS is approximately 19.2%, significantly higher than the 6%-10% observed in the general population. The risk is further increased in cases of complete outflow obstruction (25%) compared to incomplete obstruction (14%). These findings support the role of menstrual outflow obstruction as a key factor in the development of endometriosis [5].

Herlyn-Werner-Wunderlich syndrome arises from early embryologic disruption involving both the Müllerian (paramesonephric) and Wolffian (mesonephric) ducts. Normally, the paired Müllerian ducts develop, migrate medially, and fuse to form the uterus and upper vagina, while the intervening septum resorbs to create a single uterovaginal canal. Concurrently, the Wolffian ducts regress in females but play a crucial inductive role in Müllerian duct development and are essential for renal formation through the ureteric bud [9].

In HWWS, early developmental arrest of 1 Wolffian duct impairs ipsilateral Müllerian duct positioning and fusion, resulting in uterus didelphys with a duplicated cervix and hemivagina. Failure of vertical fusion with the sinovaginal bulb leads to obstructed hemivagina or cervicovaginal atresia. In this case, the absence of the left Wolffian duct explains the ipsilateral renal agenesis, while defective fusion resulted in a noncommunicating left uterine unit with outflow obstruction [3]. This correlates with imaging findings of uterus didelphys, left-sided obstruction with hematometra, and absence of the left kidney.

Persistent outflow obstruction promotes retrograde menstruation, predisposing to endometriosis, as evidenced by the presence of a left ovarian endometrioma in this patient. These findings highlight the direct link between embryologic maldevelopment and the clinical-radiologic manifestations observed, as well as the increased risk of complications in cases with complete obstruction.

A comprehensive classification of Herlyn-Werner-Wunderlich syndrome was proposed by Zhu et al. in 2015, dividing the syndrome into 4 subtypes according to the presence of a vaginal septum and the presence or absence of communication between the endometrial cavities [10]. The classification system is presented in Table 1.

**Table 1:** Classification of Herlyn-Werner-Wunderlich syndrome based on Zhu et al [10].

Classification	Subtype	Description	Outflow/Communication	Clinical presentation	Complications
Classification 1 (Completely obstructed hemivagina)	1.1 Blind hemivagina	Complete obstruction of the hemivagina; the uterus behind the septum is completely isolated; no communication between duplicated uterus and vagina	No outflow; no communication	Early onset after menarche; acute abdominal pain, fever, vomiting	Hematocolpos, hematometra, hematosalpinx, hemoperitoneum; endometriosis; pelvic adhesions; infection (pyosalpinx, pyocolpos)
	1.2 Cervicovaginal atresia without communicating uteri	Complete obstruction with atretic or maldeveloped cervix; no communication between the duplicated uteri; menstrual outflow is obstructed	No outflow; no communication	Similar to classification 1.1; early severe dysmenorrhea	Hematometra; increased risk of endometriosis
Classification 2 (Incompletely obstructed hemivagina)	2.1 Partial reabsorption of the vaginal septum	Partial communication between the 2 vaginas; the uterus behind the septum remains isolated	Limited outflow via small vaginal communication	Delayed onset, cyclic pain, purulent, or bloody vaginal discharge	Ascending genital infection
	2.2 Communicating uteri	Complete vaginal obstruction with small communication between duplicated cervices; partial drainage occurs.	Limited outflow via cervical communication	Delayed presentation; intermittent symptoms due to impaired drainage	Persistent obstruction-related complications

**Figure 7:**
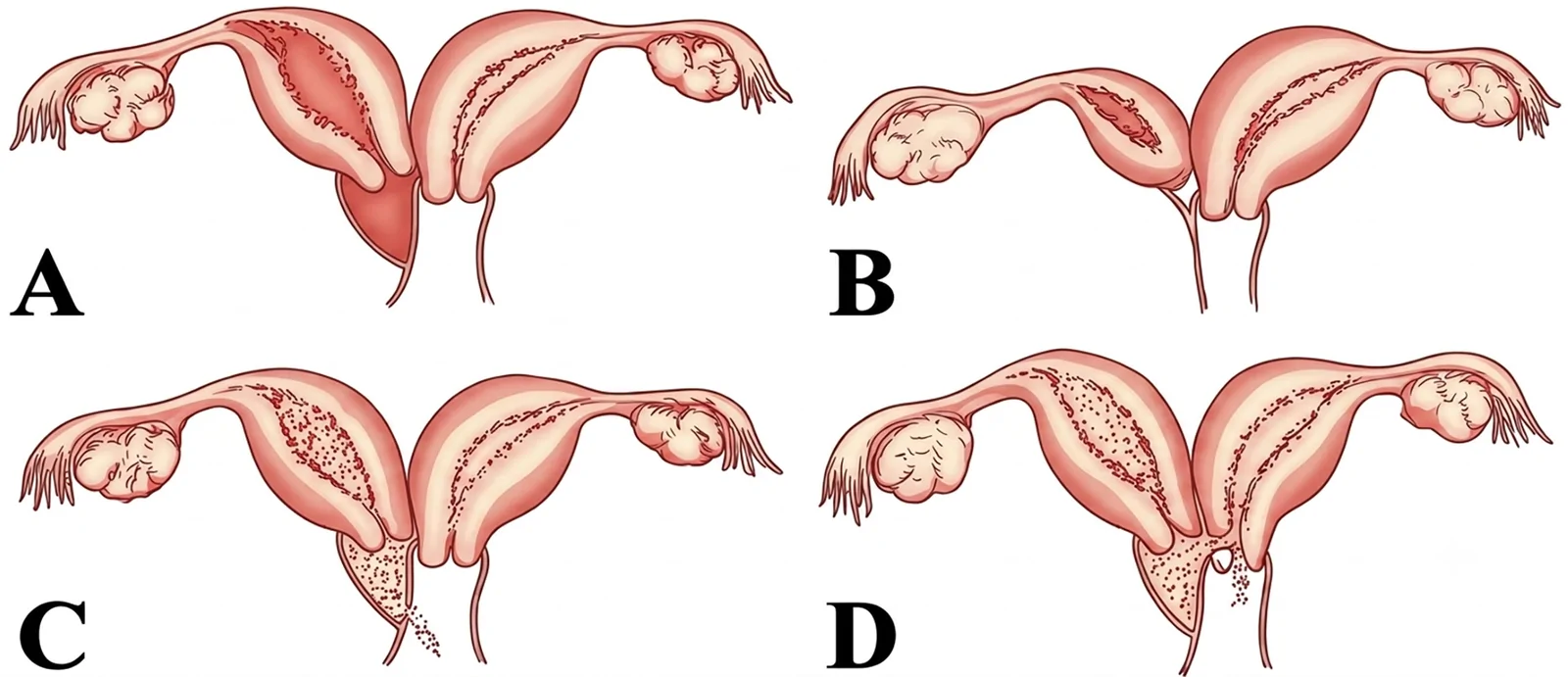
Schematic illustration of Herlyn-Werner-Wunderlich syndrome classification. (A) Classification 1.1 (blind hemivagina): complete obstruction of 1 hemivagina with no communication between the duplicated uteri or vaginas, resulting in accumulation of menstrual blood within the obstructed side. (B) Classification 1.2 (cervicovaginal atresia without communicating uteri): complete obstruction associated with an atretic or maldeveloped cervix, preventing menstrual outflow from the affected uterine unit; this subtype corresponds to the present case. (C) Classification 2.1 (partial reabsorption of the vaginal septum): incomplete obstruction due to a small communication between the hemivaginas, allowing limited menstrual drainage. (D) Classification 2.2 (communicating uteri): complete vaginal obstruction with a small communication between duplicated cervices, permitting partial outflow through the contralateral side.

To further illustrate the anatomical variations, the schematic representations of each classification are presented in Fig. 7. Herlyn-Werner-Wunderlich syndrome is categorized based on the degree of obstruction and the presence of communication between the duplicated genital tracts. In classification 1 (Fig. 7A and B), complete obstruction results in the absence of menstrual outflow, leading to early symptom onset and accumulation of blood products, such as hematocolpos and hematometra. In contrast, classification 2 (Fig. 7C and D) demonstrates partial communication, allowing limited drainage and consequently a delayed and often less acute clinical presentation [10].

In the present case, the findings are consistent with classification 1.2 (Fig. 7B), characterized by cervicovaginal atresia without communication between the duplicated uteri. This complete outflow obstruction accounts for the patient’s progressive dysmenorrhea and the MRI findings of hematometra. Furthermore, chronic retention of menstrual blood predisposes to retrograde menstruation, which likely contributed to the development of a left ovarian endometrioma in this patient. The schematic illustrations complement the radiologic findings by clarifying the underlying anatomical configuration and pathophysiological mechanism, thereby facilitating accurate diagnosis and classification.

Pelvic ultrasonography is commonly applied as an initial imaging modality due to its accessibility, cost-effectiveness, and lack of ionizing radiation; however, its diagnostic performance is operator-dependent. Ultrasound can identify key findings such as hematocolpos, hematometra, duplicated uterine cavities and vaginal canals, and associated renal agenesis [11]. Hematometra, typically appearing as a fluid collection with echogenic material, may facilitate recognition of an underlying genitourinary anomaly such as uterus didelphys. However, ultrasound has limited capability in accurately characterizing the specific type of Müllerian duct anomaly [4]. In contrast, magnetic resonance imaging (MRI) is considered the gold standard, offering superior soft-tissue resolution and a wide field of view. Its multiplanar and multiparametric capabilities enable precise characterization of Müllerian duct anomalies and related complications, including hematosalpinx and pelvic endometriosis, thereby playing a crucial role in accurate diagnosis and guiding clinical management [1,11].

T2-weighted sequences constitute the cornerstone of pelvic MRI due to their superior ability to delineate uterine zonal anatomy. Multiplanar T2-weighted imaging, including axial planes and sequences aligned with the long axis of the uterus, is essential for accurate evaluation of uterine morphology, external contour, adnexal structures, and classification of Müllerian duct anomalies. Additional axial or coronal T2-weighted imaging of the abdomen facilitates assessment of associated urinary tract anomalies. T1-weighted sequences, particularly with fat suppression, complement this evaluation by enhancing tissue contrast and enabling detection of blood products, which is crucial for identifying subacute blood products in conditions such as hematometra or hematocolpos, as well as for characterizing endometriotic lesions, including deep infiltrative endometriosis [12].

Although MRI is considered the imaging modality of choice for evaluating Müllerian duct anomalies because of its excellent soft-tissue contrast and multiplanar capability, assessment of the urinary tract remains equally important, given the close embryologic relationship between the Müllerian and mesonephric (Wolffian) ducts. CT urography provides comprehensive evaluation of the kidneys, ureters, and collecting system, allowing confirmation of associated urinary tract anomalies that are frequently encountered in OHVIRA syndrome. In the present case, CT urography demonstrated complete absence of the left kidney and left ureter while confirming normal morphology and preserved excretory function of the contralateral urinary tract. These findings complemented the MRI examination and strengthened the radiologic diagnosis by confirming the ipsilateral renal anomaly characteristic of OHVIRA syndrome [13].

The differential diagnosis of adolescents presenting with progressive dysmenorrhea and hematometra includes other Müllerian duct anomalies, such as a unicornuate uterus with a noncommunicating functional rudimentary horn, bicornuate uterus, transverse vaginal septum, cervical agenesis, and imperforate hymen. However, these entities can be distinguished based on their characteristic imaging features [14]. In the present case, MRI demonstrated 2 completely separate uterine bodies consistent with uterus didelphys, complete obstruction of the left hemivagina associated with cervical atresia, and ipsilateral renal agenesis, constituting the characteristic triad of Herlyn-Werner-Wunderlich syndrome. Unlike a unicornuate uterus with a noncommunicating horn, both uterine units in this patient were fully developed and separated. A bicornuate uterus typically demonstrates a single cervix with partial fusion of the uterine horns, whereas the present case showed complete uterine duplication. Furthermore, isolated obstructive anomalies, such as transverse vaginal septum or imperforate hymen, do not occur in association with uterus didelphys and ipsilateral renal agenesis [14,15]. The multiplanar capability and superior soft-tissue contrast of MRI enabled precise delineation of the reproductive tract anatomy and associated urinary tract anomaly, allowing confident differentiation from other Müllerian duct anomalies and accurate classification of OHVIRA syndrome [15]. This case highlights the pivotal role of MRI not only in establishing the diagnosis but also in accurately differentiating OHVIRA syndrome from other obstructive Müllerian anomalies, thereby facilitating appropriate classification and management.

An endometrioma is a hemorrhagic ovarian cyst lined by ectopic endometrial tissue, typically characterized on ultrasound by homogeneous low-level internal echoes, producing a “ground-glass” appearance. The presence of peripheral punctate echogenic foci, thought to represent cholesterol deposits or hemosiderin-related byproducts from prior hemorrhage, further increases diagnostic specificity [16]. In this case, the left adnexal lesion demonstrated typical sonographic features of an endometrioma, including homogeneous low-level echoes and peripheral punctate echogenic foci, which were confirmed on MRI by T1 hyperintensity and T2 shading. In patients with HWWS, outflow obstruction leads to increased intraluminal pressure and prolonged retention of menstrual blood, thereby promoting retrograde flow through the fallopian tubes into the pelvic cavity. This refluxed menstrual fluid contains viable endometrial cells capable of implantation and proliferation on peritoneal and ovarian surfaces [17]. Repeated cyclic bleeding within these ectopic implants results in progressive accumulation of hemorrhagic content, ultimately forming ovarian endometriomas characterized by cystic lesions filled with altered blood products [4]. In addition, chronic inflammation, immune dysregulation, and oxidative stress contribute to lesion persistence and progression [18]. These mechanisms are consistent with the imaging findings in the present case, which demonstrate hematometra and a left ovarian endometrioma, reflecting the consequences of longstanding outflow obstruction and delayed diagnosis.

The primary goal of management is to relieve the obstructive anomaly, thereby alleviating symptoms and improving future reproductive potential [19]. Definitive management of HWWS is primarily surgical, most commonly through excision of the obstructing vaginal septum [12]. A single-stage vaginoplasty involving drainage of the obstructed compartment and excision of the vaginal septum is considered the standard treatment. Various surgical approaches have been described, including laparotomic, laparoscopic, and transvaginal techniques, all aimed at relieving outflow obstruction and alleviating pain caused by retained menstrual blood. Among these, the transvaginal approach is most commonly employed, with techniques ranging from conventional sharp dissection to hysteroscopic and resectoscopic methods using monopolar or bipolar electrocautery, as well as CO₂ laser-assisted resection [20]. This intervention not only relieves obstruction but also reduces the risk of complications, particularly pelvic endometriosis, by preventing ongoing retrograde menstruation [12].

In cases where surgical intervention is not immediately feasible in patients with Herlyn-Werner-Wunderlich syndrome, hormonal therapy aimed at menstrual suppression, such as combined oral contraceptives, may be utilized to reduce further accumulation of hematocolpos and prevent progression of hematometra [4]. In patients with endometriosis, progestin therapy such as Dienogest is effective in alleviating pelvic pain and suppressing endometrial activity through antiproliferative and anti-inflammatory effects. It promotes a hypoestrogenic, decidualized endometrial environment, thereby inhibiting the growth of ectopic endometrial tissue. Clinical evidence has demonstrated its efficacy in reducing dysmenorrhea and decreasing the size of endometriotic lesions, including ovarian endometriomas [21,22]. In the present case, dienogest was administered as a conservative approach because of the patient’s preference to defer surgical intervention. This resulted in symptomatic improvement, particularly in reducing pelvic pain, likely related to suppression of endometrial activity and control of associated endometriosis. However, it does not correct the underlying obstructive anomaly associated with Herlyn-Werner-Wunderlich syndrome. Therefore, definitive surgical management remains necessary to prevent recurrence and long-term complications.

This case provides educational value by demonstrating the complementary role of multimodality imaging in the comprehensive evaluation of Herlyn-Werner-Wunderlich syndrome. While ultrasonography served as the initial imaging modality, MRI accurately delineated the complex Müllerian duct anomaly, identified the uncommon classification 1.2 variant (cervicovaginal atresia without communicating uteri), and detected associated complications, including hematometra and ovarian endometrioma. CT urography further confirmed the ipsilateral renal anomaly, completing the characteristic radiologic spectrum of the syndrome. Recognition of these imaging features is essential for accurate diagnosis, appropriate classification, timely management, and prevention of long-term complications such as endometriosis and infertility.

A limitation of this report is the absence of surgical and histopathological confirmation, as definitive surgical management had not yet been performed at the time of reporting. Nevertheless, the diagnosis was established with a high degree of confidence based on the characteristic multimodality imaging findings, including ultrasonography, MRI, and CT urography, which demonstrated the classic triad of OHVIRA syndrome and its associated complications.

## Conclusion

Herlyn-Werner-Wunderlich syndrome should be considered in adolescent females presenting with progressive dysmenorrhea, particularly when symptoms are persistent or atypical. This case highlights a type 1.2 obstructive variant with cervicovaginal atresia, in which delayed diagnosis led to secondary complications, including hematometra and ovarian endometrioma. Pelvic ultrasonography serves as an effective first-line imaging modality, whereas MRI is the gold standard for definitive diagnosis, accurate classification, and assessment of associated complications. CT urography complements MRI by providing detailed evaluation of concomitant urinary tract anomalies. Early recognition of this condition is crucial to prevent complications related to chronic outflow obstruction and retrograde menstruation. Although conservative management with Dienogest provided symptomatic relief in this patient, it does not address the underlying structural anomaly. Definitive surgical correction is therefore required to relieve obstruction, prevent recurrence, and preserve future reproductive function.

## Patient consent

A written informed consent for case publication was obtained from the patient. A copy of the completed consent form would be available on request for review by the Editor-in-Chief of this journal.
